# Supplementary material for: The interactome of intact mitochondria by cross-linking mass spectrometry provides evidence for coexisting respiratory supercomplexes
Source: Mol Cell Proteomics. 2017 Dec 8;17(2):216–32. doi: 10.1074/mcp.RA117.000470 (PMC5795388; doi:10.1074/mcp.RA117.000470)
Supplement: Supplemental Data [file supp_RA117.000470_133922_0_supp_23978_fzffwf.zip › spectra_annotation/mito_DR_spectra_annotation/49-1-5-1-16-1.pdf]

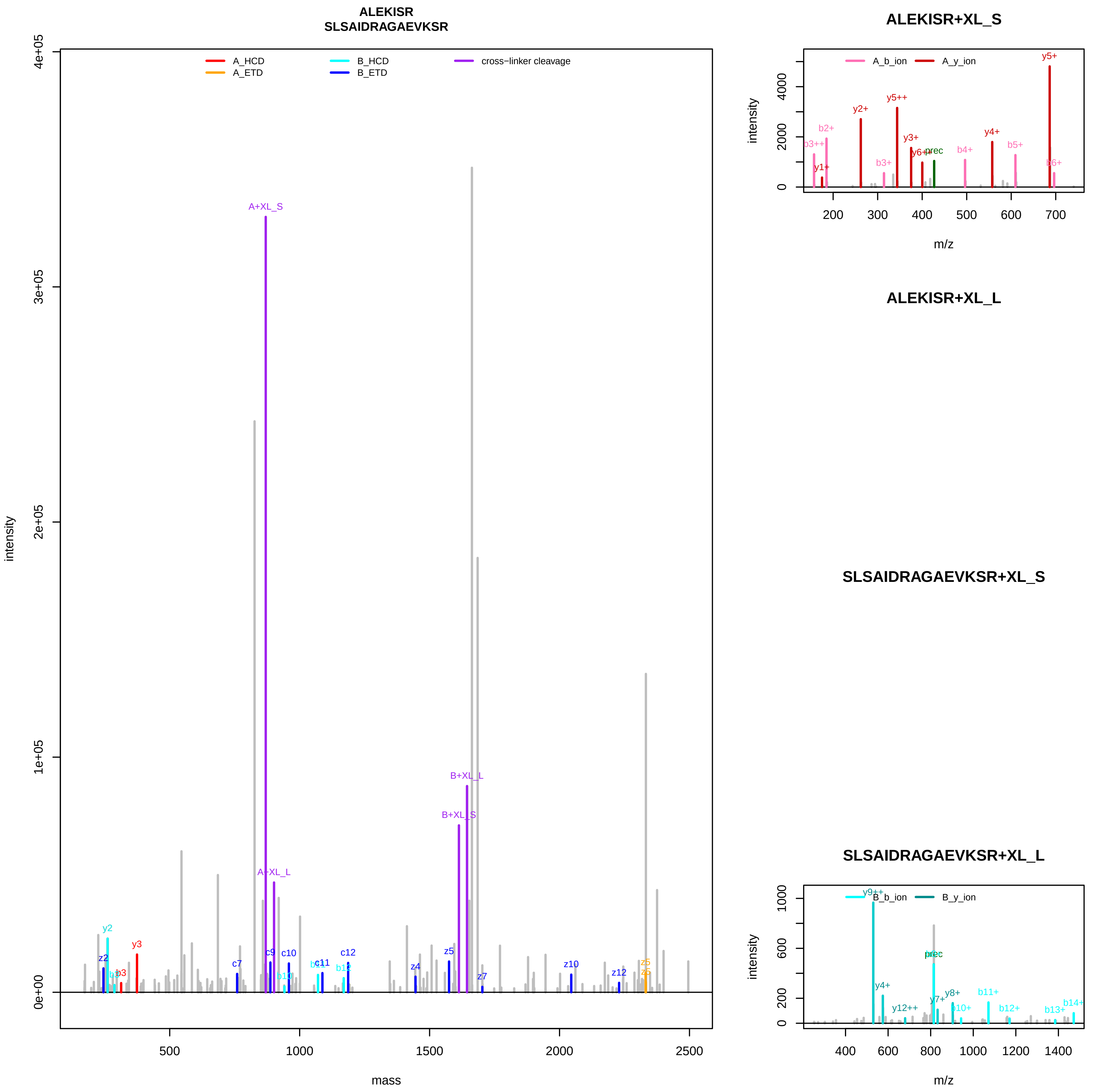
ALEKISR
SLSAIDRAGAEVKSR
A_HCD
A_ETD
B_HCD
B_ETD
cross−linker cleavage
intensity
mass
4e+05
3e+05
2e+05
1e+05
0e+00
500
1000
1500
2000
2500
A+XL_S
A+XL_L
B+XL_L
B+XL_S
y2
y3
b3
z2
c7
c9
c10
b10
c11
b11
c12
b12
z4
z5
z7
z10
z12
z5
ALEKISR+XL_S
A_b_ion
A_y_ion
y5+
y5++
y2+
b2+
b3++
y1+
b3+
y3+
y6++
prec
b4+
y4+
b5+
b6+
m/z
0
2000
4000
200
300
400
500
600
700
ALEKISR+XL_L
SLSAIDRAGAEVKSR+XL_S
SLSAIDRAGAEVKSR+XL_L
B_b_ion
B_y_ion
y9++
prec
y4+
y12++
y7+
y8+
b10+
b11+
b12+
b13+
b14+
0
200
600
1000
400
600
800
1000
1200
1400
